# Nocturnal Hypoglycaemia in Patients with Diabetes Mellitus: Database Analysis of a Cohort Using Telemedicine Support for Self-Monitoring of Blood Glucose over a 10-Year-Long Period

**DOI:** 10.3390/medicina57020167

**Published:** 2021-02-14

**Authors:** Gyorgy Jermendy, Agnes Kecskes, Attila Nagy

**Affiliations:** 1 Bajcsy-Zsilinszky Teaching Hospital and Outpatient Clinic, 89–91 Maglodi ut, 1106 Budapest, Hungary; 2 77 Elektronika Kft, 98 Fehervari ut, 1116 Budapest, Hungary; akecskes@e77.hu (A.K.); anagy@e77.hu (A.N.)

**Keywords:** diabetes management, health hazard, hypoglycaemia, real-world data

## Abstract

*Background and Objectives*: In patients with diabetes mellitus, hypoglycaemic episodes, especially during night hours, carry a significant risk. Data about the occurrence of nocturnal hypoglycaemia in real-world settings are of clinical importance. The aim of our study was to evaluate the occurrence of nocturnal hypoglycaemia among patients with diabetes using self-monitoring of blood glucose (SMBG) with telemedicine support. *Materials and Methods*: We retrospectively analysed the central database of an internet-based supportive system between 2010 and 2020 when 8190 SMBG users uploaded nearly 10 million capillary blood glucose values. Nocturnal hypoglycaemia was defined as capillary blood glucose < 3.0 mmol/L measured between 00:00 and 05:59 h. *Results*: The database contained 914,146 nocturnal blood glucose values from 7298 users; 24,623 (2.7%) glucose values were below the hypoglycaemic threshold and 2363 patients (32.4%) had at least one hypoglycaemic glucose value. Nocturnal hypoglycaemia was more often found in patients with type 1 vs. type 2 diabetes (*n* = 1890 (80.0%) vs. *n* = 387 (16.4%), respectively). Hypoglycaemic blood glucose values were most frequently observed in the age group of 10.0–19.9 years (*n* = 481 (20.4%)). Patients with nocturnal hypoglycaemia were mostly on insulin treatment (1854 (78.5%) patients with 20,727 (84.1%) hypoglycaemic glucose values). Only 356 patients (15.1%) with nocturnal hypoglycaemia performed a retest within 120 min. Within a one-day-long (1440 min) timeframe, the elapsed median time until a retest, yielding a safe blood glucose value (>3.9 mml/L), was 273 min (interquartile range: 157–300 min). *Conclusions*: Nocturnal hypoglycaemia should be considered as a persisting challenge to antihyperglycaemic treatment in patients living with diabetes. Continuous efforts are needed to improve both antihyperglycaemic treatment and patient education for preventing nocturnal hypoglycaemia, and to act adequately if hypoglycaemic values are detected.

## 1. Introduction

Hypoglycaemia carries a significant risk and often serves as a barrier for achieving optimal glucose control in patients with diabetes mellitus [1,2,3]. Patients with type 1 diabetes may experience clinical signs and symptoms of lower blood glucose values during the life-long course of diabetes [4]. In patients with type 2 diabetes, hypoglycaemia may also occur, particularly with insulin or sulfonylurea treatment [5,6,7,8,9,10]. Risk factors of hypoglycaemia in patients with diabetes are well characterized [8]. The clinical symptoms of hypoglycaemia may vary individually; cases from mild to severe hypoglycaemia may occur but blood glucose values in the hypoglycaemic range may be observed even in symptomless patients.

Self-monitoring of blood glucose (SMBG) is useful for patients with diabetes, both in self-management and for detecting hypoglycaemic glucose values [11,12]. The usefulness of SMBG may be increased by using innovative technologies, such as automatic transmission of SMBG values using a telehealth unit or mobile phone [13,14,15]. For this reason, the Dcont^®^ eNapló (eDiary) system was introduced in Hungary in 2010. This telemedicine support has been continuously available for patients and managing physicians; the central database contained nearly 10 million blood glucose values in 2020.

Nocturnal hypoglycaemia is particularly important in patients with diabetes as the clinical consequences of lower blood glucose values may be even more serious than those observed daytime. Although nocturnal hypoglycaemic episodes are regularly registered and published—with other outcome parameters—in randomized, controlled clinical trials conducted in patients with type 1 or type 2 diabetes, real-world data about their occurrence are limited [16,17]. As SMBG has increasingly become popular in daily clinical practice, important real-world aspects could be expected from an analysis of a large database of patients using SMBG regularly.

Therefore, the aim of our study was to analyse the central database of the internet-based supportive system for SMBG users in Hungary, with a special focus on nocturnal blood glucose values in the hypoglycaemic range (<3.0 mmol/L).

## 2. Materials and Methods

The Dcont^®^ eDiary (Budapest, Hungary) (available at Dcont.hu) is an internet-based telecommunication method for improving care of patients with diabetes using regular SMBG. Subjects—after registration on the website—can upload their capillary blood glucose values to the central server, via internet or mobile phone. Different statistics, graphics, mean values, and tendencies over time can automatically be generated and saved to the online server. All data of a predefined timeframe can electronically be sent to the patient for printing (Dcont^®^ eDiary) or will be available to the managing doctor—with a permission from the patient to his/her physician. This telemedicine support was introduced in Hungary in 2010. It became popular among patients as immediately available graphics and statistics facilitate the care of patients with diabetes and it is also useful for the general practitioners and specialists as they can have a look at the data and even a teleconsultation can be performed. Importantly, this internet-based telemedicine support is free of charge for the patients and available nationwide. We retrospectively analysed the capillary blood glucose values in the central database over a 10-year period (from 16 July 2010 to 15 February 2020).

Only patients with at least 10 uploaded values were involved in the analysis. The central server registered the actual blood glucose values with the exact time of each measurement. Each patient at the time of the registration (at first upload) on the website (Dcont.hu) voluntarily provided basic clinical data (age, gender, age at diagnosis of diabetes, type of diabetes, and treatment of diabetes (class of drugs only)) and agreed on the fact that the data will be stored on a central server, keeping personal anonymity. In our analysis, we followed the Helsinki Declaration and provided non-identifiable data only. Our investigation met all the requirements prescribed by the GDPR (General Data Protection Regulation).

During the nearly 10-year-long investigation period, the Dcont^®^ (abbreviation came from diabetes control) glucometers have been developed and renewed over time, both in appearance and in technical capacity. The detection limits of the glucose measurement have also been modified. In the early devices (Dcont^®^ PERSONAL, Dcont^®^ START, Dcont^®^ OPTIMUM, Dcont^®^ OPTIMUM PLUS, and Dcont^®^ PARTNER), the lower detection limit was 1.1 mmol/L whereas this was 0.6 mmol/L in the new devices (Dcont^®^ IDEAL, Dcont^®^ TREND, Dcont^®^ HUNOR, Dcont^®^ MAGOR, Dcont^®^ ETALON, Dcont^®^ NOVUM); in case of a glucose value below the lower detection limit, a “Low” signal appeared in the display. In the early devices, the upper detection limit was 25.5 or 30.5 mmol/L, whereas this was 33.3 mmol/L in the new devices; in case of a glucose value higher than the upper detection limit, a “High” signal appeared in the display. In our final analysis, we used the numerical values of the blood glucose measurements, and therefore the “Low” and “High” measurement rankings were not taken into consideration. From 2013 onwards, the glucometers displayed sufficient analytical quality, which met the more stringent accuracy criteria according to ISO 15197/2013 [18], harmonized as EN ISO 15197/2015.

Nocturnal hypoglycaemia was defined based on a glucose measurement between 00:00 and 05:59 h with a blood glucose value < 3.0 mmol/L. Although glucometers measured capillary blood glucose value, it was converted to venous plasma glucose value at displaying. In our analysis, we evaluated the occurrence of nocturnal blood glucose values in the hypoglycaemic range (*n*, %) and that of patients (*n*, %) with at least one glucose value indicating nocturnal hypoglycaemia. We assessed the distribution of nocturnal hypoglycaemia according to the age of patients and type and treatment of diabetes. We also evaluated the number of nocturnal hypoglycaemias per patient; however, we could not assess its frequency in calendar periods. In other words, we report the prevalence of nocturnal hypoglycaemia in terms of the proportions of participants with nocturnal hypoglycaemia, rather than rates of events per patient-year. Finally, we collected data about the duration of nocturnal hypoglycaemia using the values of retest (consecutive capillary blood glucose values after the first nocturnal hypoglycaemia).

In the central registry, no data were available about the clinical conditions of nocturnal hypoglycaemia, meaning that it remained unknown whether the registered hypoglycaemia was symptomless, mild, or severe. In addition, clinical or laboratory data (comorbidities, complications, clinical outcome, renal parameters, HbA1c values, etc.) were not available in the database.

We used descriptive analysis regarding the occurrence of nocturnal hypoglycaemia. Mean values with the standard deviation for the continuous parameters (age of patients) and median values with the interquartile range (IQR) for the non-parametric data (blood glucose values) are reported. Differences in continuous parameters were evaluated using Student’s unpaired test. A *p* value of <0.05 was considered statistically significant.

## 3. Results

### 3.1. Basic Characteristics of the Cohort and Frequency of Nocturnal Hypoglycaemia

During the entire investigation period, 8190 regular users (men: 5552, women: 2638) uploaded 9,867,919 blood glucose values. The number of blood glucose values below the lower detection limit (“Low” value) was 5357 (0.05%) while that of higher than the upper detection limit (“High” value) was 3286 (0.03%), resulting in 9,859,276 (99.92%) total numerical values of blood glucose, serving as the target of our further analysis.

For the nocturnal period (00:00–05:59 h), the database contained 914,146 blood glucose values from 7298 users. The number of blood glucose values within the hypoglycaemic range was 24,623 (2.7%); 2363 patients (32.4%) had at least one glucose value in the hypoglycaemic range (<3.0 mmol/L) while 4935 patients (67.6%) did not have any nocturnal hypoglycaemic glucose values. In patients with nocturnal hypoglycaemia (*n* = 2363), the age of males (*n* = 1490 (63.1%)) was significantly higher than that of females (*n* = 873 (36.9%)) (age: 35.2 ± 19.4 vs. 30.5 ± 19.3 years; *p* < 0.05).

The occurrence of nocturnal hypoglycaemia per patient ranged from 1 to 49 in most patients (533–1106 (22.6–46.8%)) with the highest occurrence of 2 to 9 cases in 1106 patients (46.8%)). Notably, a higher occurrence (50 to 99, 100 to 199, and ≥200 cases) was relatively seldom found (63, 29, and 2 patients (2.9%, 1.0%, and 0.1%), respectively) (Table 1).

### 3.2. Nocturnal Hypoglycaemia in Different Age Groups

Nocturnal hypoglycaemia was observed in each age group (Table 2). Hypoglycaemic blood glucose values were most frequently observed in the age group of 10.0–19.9 years in both genders (male: 285 (19.1%); female: 196 (22.5%); total: 481 (20.4%)). Although the occurrence of nocturnal hypoglycaemia decreased continuously with increasing age after 30 years, nocturnal hypoglycaemia was still documented among elderly people (in age-group 70.0–79.9 years: 71 patients (3.0%); and in age-group ≥80.0 years: 13 patients (0.6%).

Regarding the age of patients at diabetes manifestation, the highest proportion of nocturnal hypoglycaemia was found in patients with diabetes manifestation at the age of 10–19 years (26.7%), but that was also high at the age of <5 and 5–9 years (10.0% and 16.9%, respectively) (Table 3).

### 3.3. Nocturnal Hypoglycaemia according to the Type of Diabetes

**Figure 1:**
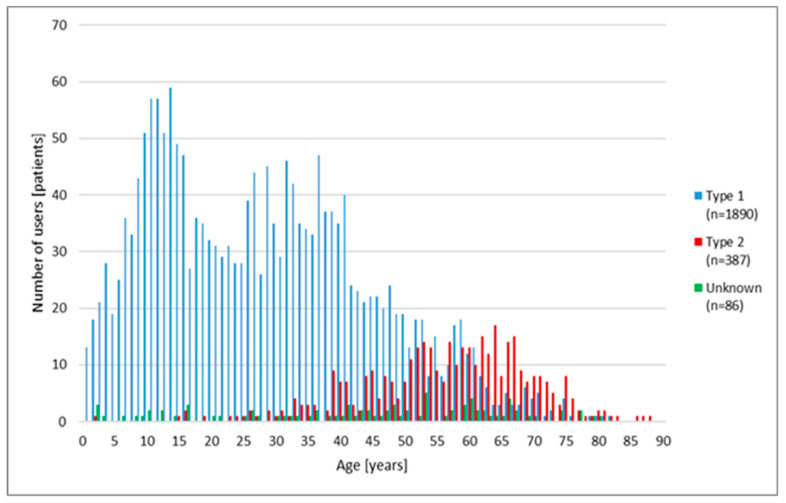
Distribution of patients (*n* = 2363) with nocturnal hypoglycaemia (at least one capillary blood glucose value < 3.0 mmol/L, between 00:00 and 05:59 h) according to the age of the patients and type of diabetes.

Among patients with nocturnal hypoglycaemia (*n* = 2363), the majority had type 1 diabetes (*n* = 1890 (80.0%)) while type 2 diabetes was documented in the minority (*n* = 387 (16.4%)); however, for 86 patients (3.6%), the type of diabetes was not provided. The distribution of patients according to age and type of diabetes is shown on Figure 1.

### 3.4. Nocturnal Hypoglycaemia in Patients with Different Treatment Strategies

We found the highest proportion (*n* = 20,727 (84.1%)) of nocturnal blood glucose values in the hypoglycaemic range in insulin-treated patients. The highest proportion of patients (*n* = 1854 (78.5%)) with nocturnal hypoglycaemia were on insulin treatment. While 209 (8.8%) patients with oral drugs + insulin treatment also had hypoglycaemic glucose values (*n* = 1405 (5.7%)), only a small number of patients had nocturnal hypoglycaemia treated either with diet only or oral drugs, similar to patients treated with non-insulin injectables. The median value of nocturnal hypoglycaemia was 2.6–2.7 mmol/L in most treatment categories, while it was 1.6 mmol/L in patients treated with oral drugs (Table 4).

### 3.5. Retest (Control Measurement) after the First Nocturnal Hypoglycaemia

Among patients with nocturnal hypoglycaemia (*n* = 2363), only 356 patients (15.1%) had at least one retest (consecutive measurement) within 120 min after the first blood glucose value in the hypoglycaemic range. In addition, 20,198 of 24,623 (82.0%) nocturnal hypoglycaemic blood glucose values were not followed by a retest within 120 min.

Within a one-day-long (1440 min) timeframe after the first nocturnal hypoglycaemia, 18,921 (76.8%) values of consecutive measurements after nocturnal hypoglycaemia were higher than the hypoglycaemia alert value (>3.9 mmol/L). The elapsing median time to retest with >3.9 mmol/L blood glucose value was 273 min (IQR: 157–300 min, total range: 1–1427 min). This median time interval was 290 min (IQR: 181–400 min, total range: 1–1427 min) when we used the retest with a higher glucose value (>6.0 mmol/L) in another analysis.

## 4. Discussion

Our database analysis provided results about nocturnal hypoglycaemia in patients with diabetes, using regular SMBG with telemedicine support. In this large cohort, nocturnal blood glucose values relatively often occurred in the hypoglycaemic range, especially in patients with insulin treatment. Children and adolescents, according to both actual age and age at diabetes manifestation, experienced more nocturnal hypoglycaemia than adults. Only a small part of patients with nocturnal hypoglycaemia performed a retest within 120 min. The elapsing median time between the first nocturnal hypoglycaemic blood glucose value and the retest, yielding a safe blood glucose value, was near to 5 h.

The central database provided a unique opportunity for analysing hypoglycaemia in a real-world setting. Our cohort consisted of patients with both type 1 and type 2 diabetes, using SMBG regularly. In Hungary, in contrast to many countries in low-resource settings [19], SMBG is available and affordable as both the test strips and devices are supported by the National Health Insurance Found for patients with insulin treatment. Costs are higher but still reasonable for patients without insulin treatment. The additional telemedicine support of SMBG has been available in the last 10 years without any extra cost.

The clinical relevance of hypoglycaemia is enormous. Hypoglycaemia is one of the leading causes of emergency hospitalization in different countries [20,21,22]. Hypoglycaemia may induce cardiac arrhythmias, which may lead to serious cardiac events [23]. The hypoglycaemia-related ECG alterations are associated with increased risk of cardiac arrhythmia, cardiovascular events, and mortality in adult patients with diabetes [24]. Nocturnal hypoglycaemia is one of the most feared complications of diabetes treatment [25,26]. The dead-in-bed syndrome is attributed, at least in some cases, to unrecognized nocturnal hypoglycaemia-induced cardiac rhythm disturbances [27,28,29]. Repeated hypoglycaemic episodes may be involved in the pathomechanism of cognitive dysfunction, both in type 1 and type 2 diabetes [30,31]. Hypoglycaemia has an impact on quality of life [32] and costs of treating hypoglycaemia are also an important concern [33].

For assessing nocturnal hypoglycaemia in our database, we used blood glucose values < 3.0 mmol/L measured between 00:00 and 05:59 h. For hypoglycaemia, a uniform definition was proposed in 2017, in which values below 3.0 mmol/L are designated as “clinically relevant” and those in the range of 3.0–3.9 mmol/L are defined as a “warning”, while a value of 3.9 mmol/L was named an alert value [34,35]. The night hours in our analysis are widely accepted for nocturnal episodes of hypoglycaemia. In this way, our results are comparable to other studies with analysis of nocturnal hypoglycaemia in patients with diabetes. 

### 4.1. Frequency of Nocturnal Hypoglycaemia

We observed nocturnal hypoglycaemia relatively frequently as 2.7% of the nocturnal measurements was within the hypoglycaemic range and 32.7% of patients with nocturnal measurements had at least one hypoglycaemic value. Nocturnal hypoglycaemia was reported from the HAT (Hypoglycaemia Assessment Tool) study, in which 40.6% of patients with type 1 diabetes and 15.9% of insulin-treated patients with type 2 diabetes had ≥1 nocturnal hypoglycaemic episode [8]. In the DIALOG study from France, 40.2% of patients with type 1 and 11.1% of patients with type 2 diabetes had nocturnal hypoglycaemia [36]. In another study from China, 16.2% patients with type 2 diabetes had nocturnal hypoglycaemia [37]. Accordingly, our results are in line with former observations regarding the frequency of nocturnal hypoglycaemia.

### 4.2. Age Groups

Nocturnal hypoglycaemia occurred in each age group; however, young people (according to both actual age and age at diabetes manifestation) were more often affected in this regard than adult people. This was probably due to insulin treatment in young people with type 1 diabetes. It is well documented that treatment of adolescents and young adults with diabetes are always challenging and a high risk of nocturnal hypoglycaemia may be considered as only one aspect of difficulties in this age-group [38].

### 4.3. Type of Diabetes

Among patients with nocturnal hypoglycaemia, the majority had type 1 diabetes (80%). Regarding patients’ distribution according to antihyperglycaemic therapy, the majority (78.5%) was only on insulin treatment while an additional part of the patients (8.8%) was treated with insulin plus oral drugs in combination. These results are consistent with former publications indicating that insulin treatment is a major risk factor for hypoglycaemia, particularly for nocturnal hypoglycaemia [2,3,8,17,36].

### 4.4. Antihyperglycaemic Treatment

Most patients with nocturnal hypoglycaemia were treated with different insulin regimes. Although basal insulin analogues (glargine U100, detemir) provide a decrease in risk of nocturnal hypoglycaemia compared to human NPH insulin [39,40], and, in addition, second generation insulin analogues (degludec, glargine U300) proved to be better than insulin glargine U100 in this respect [41,42], the prevalence of nocturnal hypoglycaemia remained an important issue. As sulfonylurea treatment may increase the risk of nocturnal hypoglycaemia, particularly among elderly patients, novel drugs (DPP-4 inhibitors, GLP-1 receptor agonists, and SGLT-2 inhibitors) with lower risk for hypoglycaemia should be preferred when deciding on the antidiabetic treatment [43,44]. Despite the availability of new antihyperglycaemic therapies, our study documented that nocturnal hypoglycaemia remained a persistent challenge in daily clinical practice.

### 4.5. Retest after Nocturnal Hypoglycaemia

After detecting nocturnal hypoglycaemia, only 15.1% of patients performed a repetitive SMBG within 120 min. While we have no data about the intervention, if any, this proportion of patients is extremely low. Moreover, the median elapsed time from the first nocturnal hypoglycaemia to the retest with glucose value in safe range was about 5 h, which should be considered long. Undoubtedly, retesting blood glucose in regular intervals (~15 min) after hypoglycaemia is fundamentally important until the blood glucose returns to normal [25]. This should be emphasized during patient education as an important element of diabetes management.

### 4.6. Limitations of the Study

Our results have to be interpreted within the context of their limitations. First, the database analysis was performed using a 10-year-long investigation period during which patients used different glucometers. Nevertheless, all devices were produced by the same company, and new glucometers from 2013 displayed sufficient analytical quality according to ISO (2013). Second, clinical conditions of nocturnal hypoglycaemic episodes were not registered in the database and, therefore, we could publish only the quantitative characteristics of these events. Third, the timeframe of nocturnal hypoglycaemia (00:00–05:59 h) is arbitrary but widely used. Fourth, the generalizability of our results is limited as patients in our study were probably well motivated with improved patient–physician communication. Fifth, only classes and not particular drugs of antihyperglycaemic agents were evaluated. Despite these limitations, we feel that our results are valuable and useful as a real-world report about nocturnal hypoglycaemia from Hungary. The large cohort with a long investigation period and using the same central database should be considered as the strengths of our study.

### 4.7. Prevention of Nocturnal Hypoglycaemia

Our data indicate that nocturnal hypoglycaemia carries a significant risk for daily clinical practice, even in recent years. Bearing this in mind, antihyperglycaemic agents with a lower risk of hypoglycaemia (in patients with type 1 diabetes: insulin analogues, especially second-generation basal insulin analogues; in patients with type 2 diabetes: SGLT-2-inhibitors, GLP-1-recepetor agonists, or DPP-4-inhibitors) should be preferred [45,46]. Importantly, the clinical significance of medical nutrition therapy should also be emphasized as dietary carbohydrate restriction could frequently lead to a reduction or elimination of different antihyperglycaemic medications with hypoglycaemic side effects [47,48]. In addition, continuous efforts are needed to improve patient education, with special focus on prevention and management of hypoglycaemia [49]. Finally, CGMS should also be indicated to recognize nocturnal hypoglycaemia, especially in patients treated with insulin or sulfonylurea [50].

## 5. Conclusions

Nocturnal blood glucose values were relatively often observed in the hypoglycaemic range in our large cohort of patients with diabetes who use the regular SMGB with telemedicine support. Nocturnal hypoglycaemia has remained a persistent challenge for antidiabetic treatment, even in recent years, indicating that further efforts are needed to decrease its occurrence in daily clinical practice.

## Figures and Tables

**Table 1:** Occurrence of nocturnal hypoglycaemia per patient.

Number of Nocturnal Hypoglycaemic Values (*n*)	Females *n* (%)	Males *n* (%)	Total *n* (%)
1	228 (26.1)	402 (27.0)	630 (26.7)
2–9	418 (47.9)	688 (46.2)	1106 (46.8)
10–49	193 (22.1)	340 (22.8)	533 (22.6)
50–99	24 (2.7)	45 (3.0)	69 (2.9)
100–199	9 (1.0)	14 (0.9)	23 (1.0)
≥200	1 (0.1)	1 (0.1)	2 (0.1)
Total	873 (100.0)	1490 (100.0)	2363 (100.0)

**Table 2:** Occurrence (*n* (%)) of nocturnal hypoglycaemia (at least one blood glucose value < 3.0 mmol/L between 00:00 and 05:59 h) in patients of different age groups.

Age-Groups (Years)	Females *n* (%)	Males *n* (%)	Total *n* (%)
<10.0	115 (13.2)	129 (8.7)	244 (10.3)
10.0–19.9	196 (22.5)	285 (19.1)	481 (20.4)
20.0–29.9	153 (17.5)	194 (13.0)	347 (14.7)
30.0–39.9	151 (17.3)	261 (17.5)	412 (17.4)
40.0–49.9	92 (10.5)	234 (15.7)	326 (13.8)
50.0–59.9	78 (8.9)	191 (12.8)	269 (11.4)
60.0–69.9	53 (6.1)	147 (9.9)	200 (8.5)
70.0–79.9	30 (3.4)	41 (2.8)	71 (3.0)
≥80.0	5 (0.6)	8 (0.5)	13 (0.6)
Total	873 (100.0)	1490 (100.0)	2363 (100.0)

**Table 3:** Occurrence (*n* (%)) of nocturnal hypoglycaemia (at least one blood glucose value < 3.0 mmol/L between 00:00 and 05:59 h) according to the age of patients at diabetes manifestation.

Age at Diabetes Manifestation (Years)	Females *n* (%)	Males *n* (%)	Total *n* (%)
<5	94 (11.7)	117 (9.0)	211 (10.0)
5–9	173 (21.6)	182 (14.0)	355 (16.9)
10–19	213 (26.6)	349 (26.8)	562 (26.7)
20–29	126 (15.7)	221 (16.9)	347 (16.5)
30–39	100 (12.5)	186 (14.3)	286 (13.6)
40–49	52 (6.5)	153 (11.7)	205 (9.7)
50–59	34 (4.2)	62 (14.0)	96 (4.6)
≥60	10 (1.2)	34 (2.6)	44 (2.1)
Total *	802 (100.0)	1304 (100.0)	2106 (100.0)

* For 257 patients, data were not available.

**Table 4:** Occurrence (*n* (%)) of nocturnal hypoglycaemia (at least one blood glucose value < 3.0 mmol/L between 00:00 and 05:59 h) in patients with different treatment strategies.

Antidiabetic Treatment	Patients *n* (%)	Blood Glucose Values, *n* (%)	Blood Glucose, Interquartile Ranges (mmol/L)				
			Min	25%	50%	75%	Max
Diet only	36 (1.5)	641 (2.6)	0.6	2.3	2.6	2.8	2.9
Oral drugs	37 (1.6)	60 (0.3)	0.6	1.1	1.6	2.6	2.9
Oral drugs + insulin	209 (8.8)	1405 (5.7)	0.6	2.3	2.6	2.8	2.9
Insulin	1854 (78.5)	20,727 (84.1)	0.6	2.4	2.7	2.8	2.9
Injectables, non-insulin	6 (0.3)	12 (0.1)	0.6	2.5	2.7	2.9	2.9
Not known	221 (9.3)	1778 (7.2)	0.6	2.3	2.6	2.8	2.9
Total	2363 (100.0)	24,623 (100.0)	0.6	2.4	2.7	2.8	2.9

## Data Availability

The original datasets are not publicly available due to data protection policies. The data presented in this study are available on scientific request from the corresponding author.
